# Two new species of *Polydesmus* Latreille, 1802/1803 from northern Spain with reinstatements of two species, and a key to the Iberian *Polydesmus* species (Diplopoda, Polydesmida, Polydesmidae)

**DOI:** 10.3897/zookeys.888.37816

**Published:** 2019-11-11

**Authors:** Per Djursvoll

**Affiliations:** 1 Department of Natural History, University Museum of Bergen, Allégaten 41, 5007 Bergen, Norway University Museum of Bergen Bergen Norway

**Keywords:** Asturias, Cantabria, cave, millipede, *
Propolydesmus
*, taxonomy

## Abstract

*Polydesmus biscayensis***sp. nov.** and *P. asturiensis***sp. nov.** are described and figured based on material housed in the Museo Nacional de Ciencias Naturales in Madrid. The specimens were collected in six localities in the Asturias and Cantabria provinces, including four caves. In addition, *Polydesmus haroi* Mauriès & Vicente, 1977 and *Polydesmus racovitzai* Brolemann, 1910 are transferred from *Propolydesmus* Verhoeff, 1895 to *Polydesmus* Latreille, 1802/1803 after examining the gonopod morphology. A key to the Iberian *Polydesmus* species is presented.

## Introduction

The Holarctic family Polydesmidae comprises of more than 240 occurring species, with 192 recorded in Europe (Kime and Enghoff 2011; Enghoff et al. 2015). Most species belong to the genera *Polydesmus* Latreille, 1802/1803 and *Brachydesmus* Heller, 1858. However, new species are detected, even in Europe, particularly in the Mediterranean region and in caves (e.g., Mauriès 2013, 2014; Antić et al. 2013a, b; Gilgado et al. 2015). From the Iberian Peninsula, Azores, Madeira, Canary Islands, and Balearic Islands, 33 species in six genera, *Polydesmus* (10 spp.), *Propolydesmus* Verhoeff, 1895 (5 spp.), *Archipolydesmus* Attems, 1898 (9 spp.), *Schizomeritius* Verhoeff, 1931 (6 spp.), *Brachydesmus* (2 spp.) and *Tolosanius* Attems, 1952 (1 sp.) are recorded (see Table 1). Notably, most of them are endemic to the Iberian Peninsula.

**Table 1.** Species of Polydesmidae in Portugal and Spain and its known distribution.

Species	Portugal	Spain	Province/region
*Archipolydesmus altibaeticus* Gilgado, Enghoff, Tinaut & Ortuño, 2015		×	Granada
*Archipolydesmus bedeli* (Brolemann, 1902)		×	Segovia, Madrid and Guadalajara
*Archipolydesmus cordubaensis* Mauriès, 2013		×	Córdoba
*Archipolydesmus foliatus* Gilgado, Enghoff, Tinaut & Ortuño, 2015		×	Alicante
*Archipolydesmus giennensis* Mauriès, 2014		×	Jaén
*Archipolydesmus osellai* Ceuca, 1968		×	Huesca
*Archipolydesmus panteli* (Brolemann, 1900)		×	Cuenca, Tarragona and Lleida
*Archipolydesmus ribauti* (Brolemann, 1926)		×	Gerona
*Archipolydesmus terreus* (Attems, 1952)		×	Cádiz and Gipuzkoa
*Brachydesmus proximus* Latzel, 1889	×	×	Madeira, Azores, Canary Islands, Balearic Islands, Huesca, Malaga
*Brachydesmus superus* Latzel, 1884	×	×	Azores, Madeira, Lisbon, Balearic Islands, Canary Islands, Granada, Orense, Pontevedra, Zamora, Burgos, Madrid, Tarragona, La Rioja, Córdoba , Segovia, Navarra, Álava, Barcelona
*Polydesmus angustus* Latzel, 1884		×	Álava, Asturias
*Polydesmus asturiensis* sp. nov.		×	Asturias
*Polydesmus biscayensis* sp. nov.		×	Asturias, Cantabria
*Polydesmus coriaceus* Porat, 1871	×	×	Widely distributed in northern Iberia and adjacent islands
*Polydesmus geochromus* Attems, 1952		×	Jaén, Sevilla
*Polydesmus haroi* (Mauriès & Vicente, 1977)		×	Zamora
*Polydesmus incisus* Brolemann, 1921		×	Pyrenees, Girona, Huesca
*Polydesmus inconstans* Latzel, 1884	×	×	Navarra, Huesca, Madrid, Orense, Pontevedra, Viana do Castelo
*Polydesmus minutulus* Mauriès & Barraqueta, 1985		×	Viscaya
*Poydesmus racovitzai* (Brolemann, 1910)		×	Gipuzkoa, Viscaya, Navarra
*Propolydesmus dismilus* (Berlese, 1891)		×	Balearic Islands, Canary Islands, Valencia, Granada, Zamora, Huesca, Salamanca, Álava, Madrid, Segovia, Cuenca, Zaragoza, Toledo, Alicante, Guadalajara, Burgos
*Propolydesmus heroldi* (Schubart, 1931)		×	Sevilla
*Propolydesmus laevidentatus* (Loksa, 1967)	×	×	Canary Islands, Orense, Pontevedra, Minho, Azores, Madeira
*Propolydesmus miguelinus* (Attems, 1908)	×		Beira Litoral, Azores, Madeira
*Propolydesmus pectiniger* (Verhoeff, 1893)	×		Beira Litoral
*Schizomeritius phantasma* (Verhoeff, 1925)		×	Madrid and Àvila
*Schizomeritius andalusis* Djursvoll, 2008		×	Sevilla, Huelva and Cadis
*Schizomeritius armatus* (Machado, 1946)	×		Beira Litoral
*Schizomeritius esgrimidor* Djursvoll, 2008		×	Àvila
*Schizomeritius mauriesi* (Vicente, 1979)		×	Caceres
*Schizomeritius ortizi* Djursvoll, 2008		×	Toledo
*Tolosanius parvus* Attems, 1952		×	Gipuzkoa

**Figure 1.**
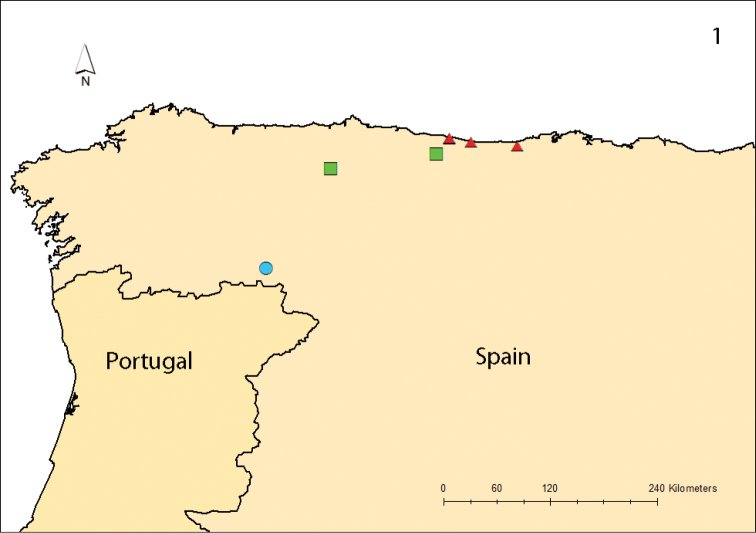
Records of *P. haroi* (Mauriès & Vicente, 1977), blue circle; *P. asturiensis* sp. nov., green square; *P. biscayensis* sp. nov., red triangle.

Two new species of *Polydesmus* are described below, based on material housed in the Museo Nacional de Ciencias Naturales, Madrid. They were collected in northern Spain, from five localities in Asturias and one in Cantabria (Fig. 1).

Both species have strongly bifurcate gonopods, with well-developed endomere (endm) and exomere (exm) (see terminology in Djursvoll 2008). Based on the length and shape of the endomere and exomere, including the length and orientation of the seminal groove, the new species are placed in the genus *Polydesmus*, as diagnosed by Djursvoll et al. (2001). Some of these character states are also present in species considered by Enghoff and Golovatch (2003) to belong in the genus *Propolydesmus*, notably *Polydesmus haroi* Mauriès & Vicente, 1977 and *Polydesmus racovitzai* Brolemann, 1910. After an in-depth examination and comparison of the gonopod morphology, both species are transferred back to *Polydesmus* in the present paper.

## Materials and methods

Preserved specimens were examined in 70 % ethanol using a Leica MZ Apo stereomicroscope. When making Scanning Electron Micrographs (SEM), structures such as gonopods and antennae were gently mounted on stubs using sticky tabs and the air-dried stubs were sputter coated with gold. A Zeiss Supra 55 UP field emission scanning electron microscope used for observation and photographs. Photographs of tergal structures were made with an Olympus SC 50 camera mounted on an Olympus SZX 10 stereomicroscope using Olympus software.

Morphological terminology for this studied group follows Djursvoll (2008). Nonetheless, some names of the gonopod structures are specified here. **Endomere**, the main gonopod part which brings the seminal groove to the solenophore – surrounded with the pulvillus. The same part is identical to “solenomere” and “femorite”, occasionally used by other authors. **Acropodite**, a tooth that originates from the distal part of endomere, always distal to the solenophore/pulvillus. The same part is identical to “distofemoral process” occasionally used by other authors. Several acropodites may occur. **Exomere**, gonopod part that originates basally on endomere, usually lateral and sometimes with a marked sulcus, usually with outgrowth of several **teeth** (processes). Abbreviations: exm = exomere, endm = endomere, t = tooth on exomere, a = acropodite.

The type material is stored in the Museo Nacional de Ciencias Naturales, Madrid (**MNCN**). New material of *Polydesmus racovitzai* Brolemann, 1910 was mainly collected during a field trip in April 2009; participants were Karin Voigtländer, Hans Reip, Norman Lindner, Helen Read, Desmond Kime, Paul Richards, Steve Gregory, and Per Djursvoll. The new material of *Polydesmus racovitzai* is stored in the University Museum of Bergen (**ZMBN**) and Senckenberg Museum of Natural History Görlitz (**SMNG**).

## Taxonomy

### Polydesmus biscayensis sp. nov.

Taxon classificationAnimaliaPolydesmidaPolydesmidae

440A2BA6-A2F0-5570-A2D6-45290F1DB56B

http://zoobank.org/82FA05D9-FF77-4FC8-88E5-21FDF3F37A7D

Figs 2–3 , 4–7 , 8

#### Type specimens.

Spain, **Asturias province**; holotype ♂; Llanes, Cueva de la Colluvina; 1 Nov. 1969; E. Ortiz leg.; MNCN 20.07/1440 • paratype ♂; same data as holotype; MNCN 20.07/2020 • paratypes 2 ♂♂, 2 ♀♀ (fragments); Llanes, Bricia, La Cueva de Tebellin; C. Cardin leg.; date unknown; MNCN 20.07/1446 • paratype ♂ (fragments in three parts); Llanes, Piedra Llanes; 26 Jan. 1929; C. Cardin leg.; MNCN 20.07/1297. **Cantabria province** • paratypes ♂, ♀, 2 juveniles (fragments); Cueva de la Busta; 7 Aug. 1968; E. Ortiz leg.; MNCN 20.07/1320.

#### Etymology.

Named after the Bay of Biscay.

#### Diagnostic characters.

Differs from other *Polydesmus* species in having a well-developed twisted endomere together with the acropodites – a1 close to the solenophore and a hooked acropodite a2 at the distalmost end, a long, slender and curly exomere, together with the presence of a ventrolateral tooth t1 directed proximal just after main curvature point, and the placement of the distal t2 tooth distally.

#### Description.

**Figures 2–3.**
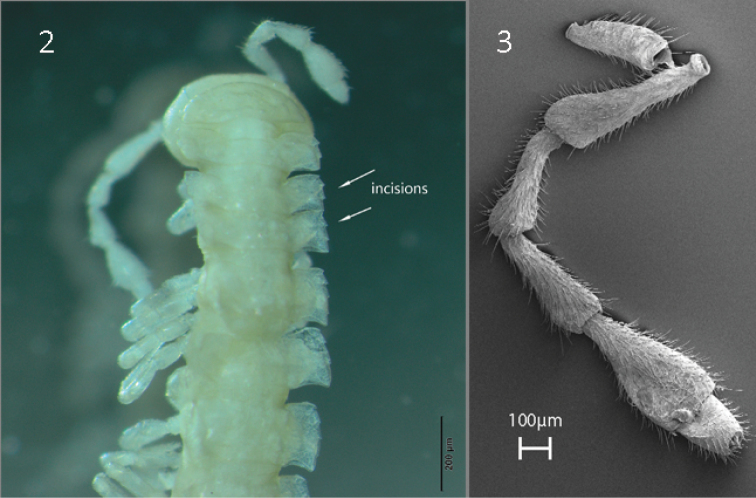
*P. biscayensis* sp. nov., male paratype **2** dorsal view of head and anterior body rings (MNCN 20.07/2020) **3** antenna (MNCN 20.07/1297).

With 20 body rings, total length 10–12 mm. Coloration whitish to pale yellow (longtime ethanol-preserved specimens only). Collum ovoid, narrower than head and the subsequent rectangular metatergum 2 which is approximately as wide as head, head > collum > metatergum 2 (Fig. 2). Antennae comparatively long, not surpassing body ring 3, antennomere 6 almost clavate and slightly longer than 4 and 5, 4 = 5 < 6 >> 7, with dorsoparabasal sensory knob on antennomere 7, sensillar area on antennomere 5–7 (Fig. 3). Metaterga rectangular, paraterga projected laterad, tergal sculpture (tuberculation) in three transverse rows, third row barely visible in metaterga 2–4. Setae clavi- to bacilliform, caudolateral part of paraterga with distinct keels especially from metarterga 4 and back. Ozopore located slightly inside caudolateral margin. Three distinct lateromarginal incisions in paratergum 2–4, four incisions on 5, incisions less distinct more posteriorly. Epiproct pointed apically. Male legs distinctly swollen, sphaerotrichomes present. Legs 1.5–2.0 times as long as midbody height, with single dorsal macrosetae on tibia.

**Figures 4–7.**
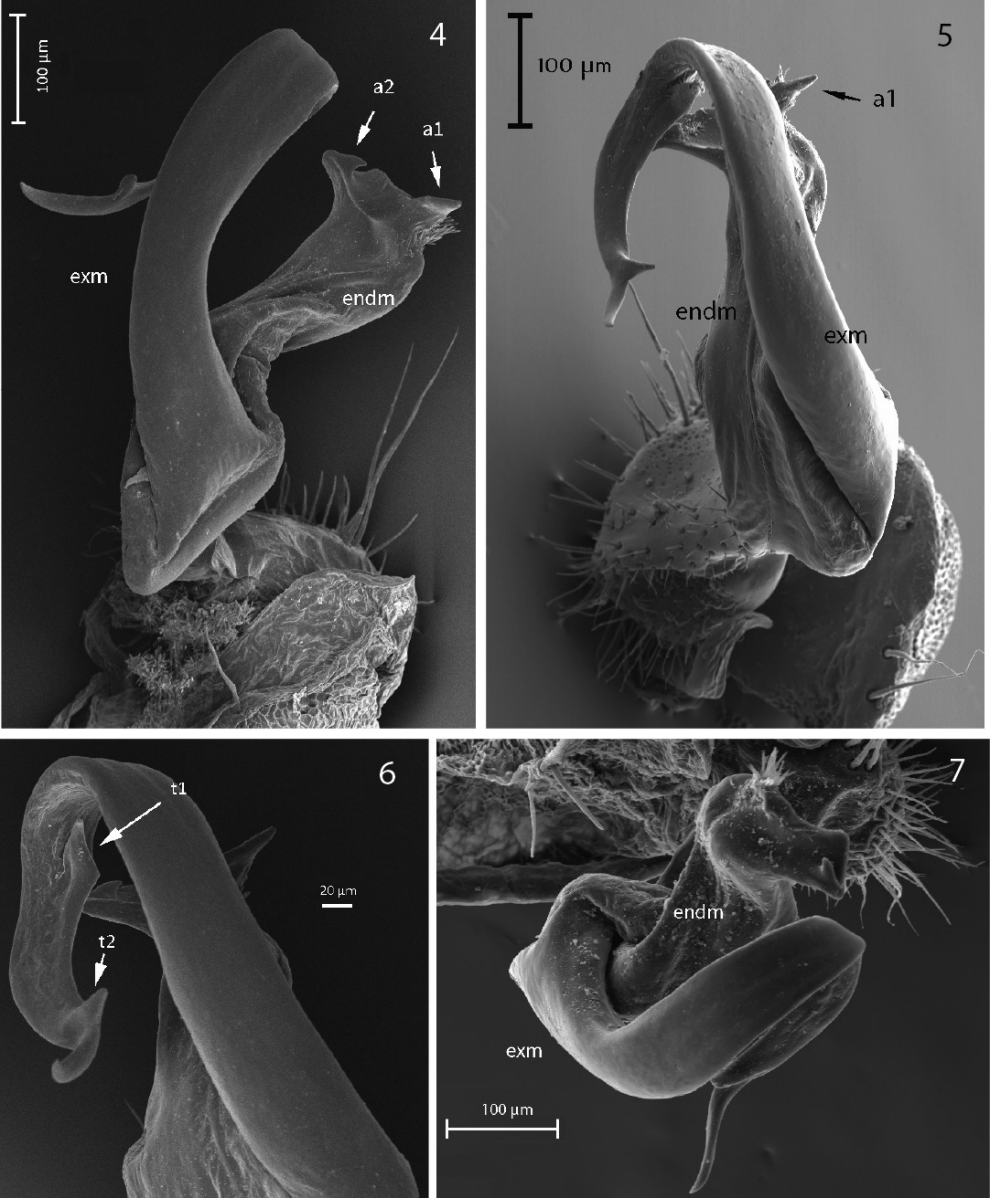
*P. biscayensis* sp. nov. **4** male paratype, right gonopod, dorsolateral view (MNCN 20.07/1297) **5** male holotype, right gonopod, dorsomesal view (MNCN 20.07/1440) **6** male paratype, right gonopod with ventrolateral tooth t1 on exomere (MNCN 20.07/1297) **7** male holotype, right gonopod, distal view (MNCN 20.07/1440).

Gonopod strongly bifurcate, including endomere and exomere, both parts twisted (curved). Endomere turns mesally crossing beneath oppositely directed exomere (Figs 4–7). Endomere stouter, bringing descending seminal groove to a mesad-directed solenophore-pulvillus surrounded with two small acropodites, a1 beside pulvillus, a2 hooked, and an excavation in between them (Fig. 4). Exomere originates from endomere with marked sulcus, very elongated and curly, descending to acute apex, with ventrolateral tooth t1 just after main curvature point directed backwards, t2 distally (Fig. 6). Prefemoral part densely setose. Lateral edge of coxite with two large macrosetae. Cannula tube-like and curved.

**Figures 8–11.**
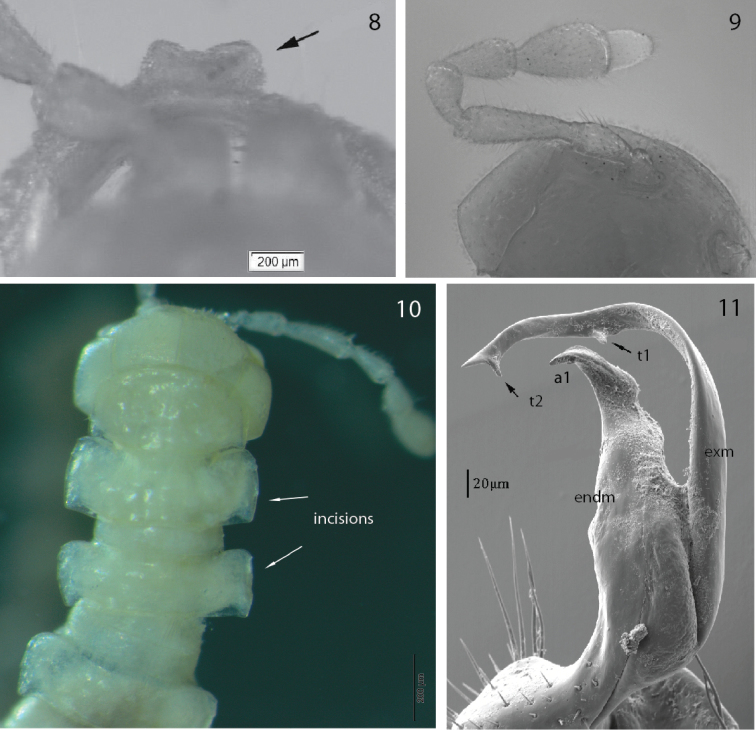
**8***P. biscayensis* sp. nov., paratype, female epigyne (MNCN 20.07/1320) **9***P. asturiensis* sp. nov., paratype, antenna (MNCN 20.07/1450) **10***P. asturiensis* sp. nov., paratype, head and anterior body rings, dorsal view (MNCN 20.07/1481) **11***P. asturiensis* sp. nov., male paratype right gonopod, mesal view (MNCN 20.07/1450).

Female with marked apophysis (tubercle) supporting the orifice of the gonopore on second coxae. Epigynal ridge poorly modified but with pin-shaped median process, with crevice inside (Fig. 8). Vulva relatively short, e.g., in lateral view less than 2× as long as high.

### Polydesmus asturiensis sp. nov.

Taxon classificationAnimaliaPolydesmidaPolydesmidae

726E0F65-24CA-55F8-8A3C-DD479DB96FA7

http://zoobank.org/91CCD20A-D879-4A5E-85FC-76550041F258

Figs 9–11 , 12–16

#### Type specimens.

Spain, **Asturias province**; holotype ♂ (fragments); Teverga, Cueva de Huerta, 750 m a.s.l.; UTM 29TQH37; July. 1934; Bolivar col.; MNCN 20.07/1484 • paratype ♂; same data as holotype; MNCN 20.07/2021 • paratypes ♂, 2 ♀♀ (fragments); same data as holotype; MNCN 20.07/1481 • paratypes ♂, ♀ (fragments); Vega de Enol; ca. 1050 m a.s.l.; 2 Nov. 1969; E. Ortiz leg.; MNCN 20.07/1450.

#### Additional material.

Two specimens in fragments, same locality data as holotype (MNCN 20.07/1484).

#### Etymology.

Named after the province of Asturias.

#### Diagnostic characters.

Differs from other *Polydesmus* species in having a twisted endomere with a distinct cleavage basad to the solenophore-pulvillus, with acute a1 distally. Exomere subfalcate, long and slender, with a ventrolateral right-angled tooth t1 just after main curvature point and together with the placement of the distal second tooth t2 close to apex.

#### Description.

With 20 body rings, total length 7–10 mm. Coloration whitish to pale yellow (long-term ethanol-preserved specimens only). Tegument shiny. Collum ovoid, much narrower than head and metaterga 2, head >> collum << metatergum 2 (Fig. 10). Antennae comparatively long, not surpassing somite 3, antennomere 6 almost clavate, slightly longer than 4 and 5, 4 = 5 < 6 >> 7. With dorsoparabasal sensory knob on antennomere 7, sensillar area on antennomere 5–7 (Fig. 9). Metaterga almost rectangular, tergal sculpture (tuberculation) in three transverse rows, third row barely visible in metaterga 2–4. Paraterga horizontal and rounded anterolaterally, paraterga 2–8 with barely visible lateromarginal incisions (not serrate), with gradually larger caudolateral projections from paraterga 5. Setae minute, barely visible. Ozopore located slightly inside caudolateral margin. Epiproct pointed apically. Male legs distinctly swollen, sphaerotrichomes present. Legs 1.5–2 times as long as midbody height, with single dorsal macrosetae on tibia.

**Figures 12–16.**
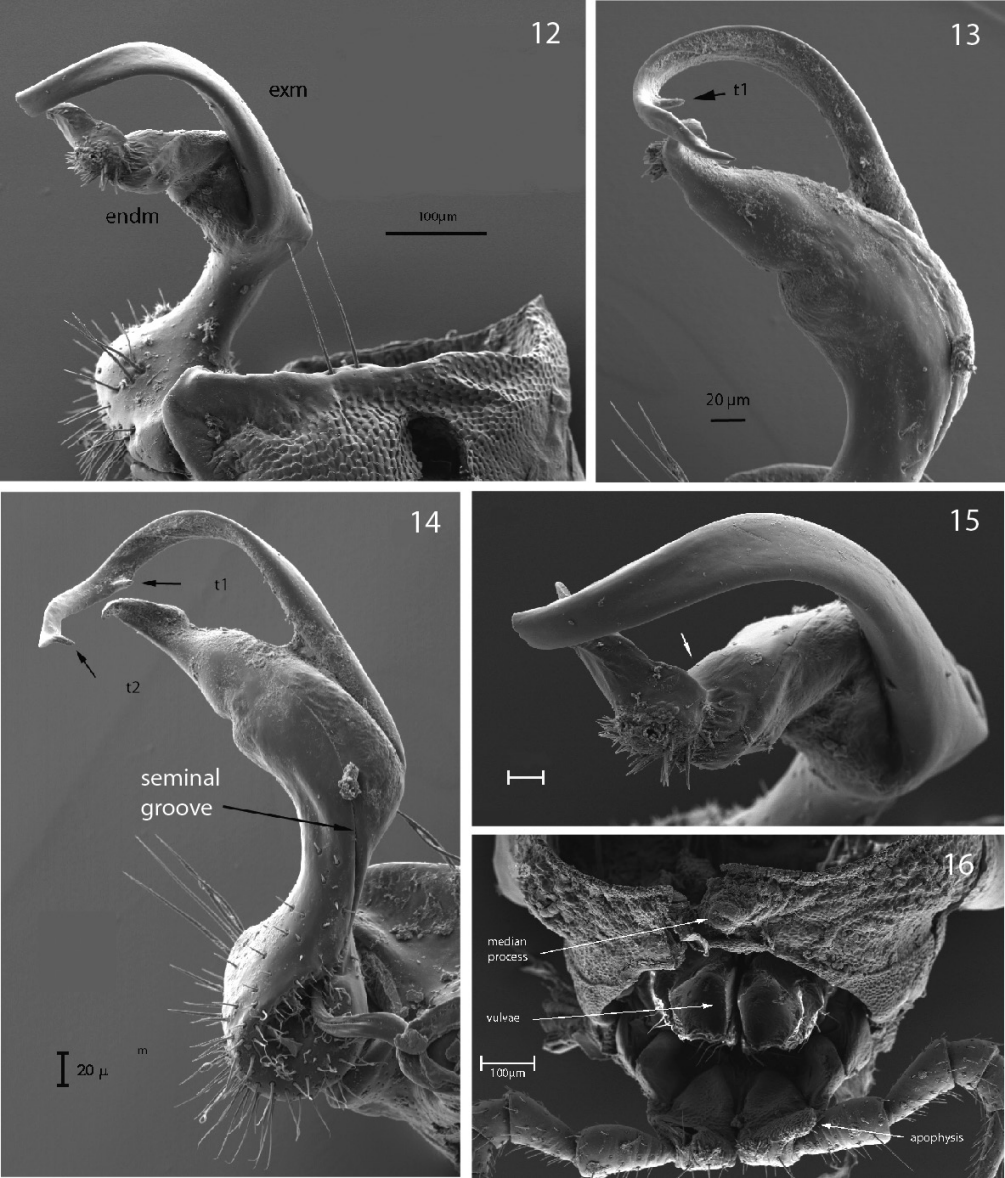
**12***P. asturiensis* sp. nov., male paratype, left gonopod, lateral view (MNCN 20.07/1481) **13–14***P. asturiensis* sp. nov., male paratype, right gonopod, mesal view (MNCN 20.07/1450) **15***P. asturiensis* sp. nov., male paratype, left gonopod, anterior view (MNCN 20.07/1481) **16***P. asturiensis* sp. nov., paratype, female epigyne and vulva (MNCN 20.07/1481).

Gonopod strongly bifurcate, including endomere and exomere (Figs 11–15). Endomere stouter, somewhat twisted, with descending seminal groove crossing beneath exomere to a distad-projecting solenophore-pulvillus, cleavage almost cut it into pieces behind solenophore-pulvillus, a1 distally smooth and pointed (Fig. 15). Exomere curved, originating from endomere with sulcus, with t1 and t2 tooth. Prefemoral part densely setose. Lateral gonocoxal edge with two large macrosetae.

Female with marked apophysis (tubercle) supporting the orifice of the gonopore on second coxae (Fig. 16). Epigynal ridge poorly modified but with pin-shaped median process, with crevice inside. Vulva relatively short, e.g., from lateral view less than 2× as long as high.

### Polydesmus haroi

Taxon classificationAnimaliaPolydesmidaPolydesmidae

Mauriès & Vicente, 1977

B4D6AE54-DE63-5CFB-8227-5E7419BD9CDB

Fig. 20

Polydesmus haroi Mauriès & Vicente, 1977: 530.
Polydesmus (Hormobrachium) haroi Vicente, 1979: 23.
Propolydesmus haroi (Mauriès & Vicente, 1977): Enghoff and Golovatch (2003: 82), Kime and Enghoff (2011: 69), Djursvoll and Melic (2015: 8).

#### Notes.

The species was figured and described in detail by Mauriès and Vicente (1977) based on material collected at Lago de Sanabria in Zamora province, with the main characters being the gonopod with two main gonopodal branches, exomere and endomere, and the seminal groove and solenophore-pulvillus extended onto endomere. These two characters differ from *Propolydesmus* but are in accordance with and support a phylogenetic relationship with the genus *Polydesmus* Latreille, 1802/03, and its type species *Polydesmus complanatus* Linnaeus, 1761.

It has similarities with *P. asturiensis* sp. nov. and P. *biscayensis* sp. nov. but differ in having a shorter exomere, the solenophore-pulvillus placed dorsally on the endomere and directed towards the exomere (Fig. 20), a wider space between the endomere and exomere, and in having a larger body size (length 17 mm). *Propolydesmus haroi* is here transferred back to *Polydesmus*.

### Polydesmus racovitzai

Taxon classificationAnimaliaPolydesmidaPolydesmidae

Brolemann, 1910

D0A455B1-5FEB-5B5A-9BBF-210447524106

Fig. 19

Polydesmus racovitzai Brolemann, 1910: 352: Attems 1927: 55, Demange 1981: 125, figs 170, 171.
Polydesmus (Hormobrachium) racovitzai Brolemann, 1910: Attems 1940: 48.
Propolydesmus racovitzai (Brolemann, 1910): Enghoff and Golovatch 2003: 82, Kime and Enghoff 2011: 69, Djursvoll and Melic 2015: 8.

#### Material examined.

SPAIN – **Viscaya province** • 1 ♂; 4 km s of Arrazua; pinewood; Desmond Kime leg.; 4.4.1978; ZMBN-ENT-PDESMID-49. – **Gipuzkoa province** • 1 ♂; Sierra de Aralar, Tolosa, 500 m south of Bedaio/Goikoa; 43.0494N, 2.04W; ca. 420 m a.s.l.; 21.4.2009; Helen Read leg.; farm buildings, under stones and logs; ZMBN-ENT-PDESMID-66 • 2 ♂♂; same collecting data as for preceding; 22.4.2009; Desmond Kime leg.; ZMBN-ENT-PDESMID-146, ZMBN-ENT-PDESMID-194)• 1 ♂; Sierra de Aralar, Beasain, road from Lazkao to Etxarri-Aranaz, west of the Pass Puerto de Lizzarusti; 42.9572N, 2.1122W; ca. 550 m a.s.l.; 21.4.2009; Voigtlander, Reip & Lindtner leg.; forest of *Fagus*, in leaf litter; SMNG-14763. – **Navarra province** • 2 ♂♂, 2 juveniles; Leitza, Ariz Mendiak, between area “Ustarleku” and “Karobieta” above side stream to Gorriztaran; 43.0778N, 1.8775W; ca. 615 m a.s.l.; 20.04.2009; Per Djursvoll leg.; grove of *Castanea*, pollard trees on the slope with *Ranunculus ficaria*, *Daphne*, *Helleborus*, *Salvia*, *Rubus*, *Lathrea*, loamy and calcareous soil, under leaves and dead wood; ZMBN-ENT-PDESMID-133, ZMBN-ENT-PDESMID-135 • 1 ♂; Leitza, town area; 43.0788N, 1.9161W; ca. 470 m a.s.l.; 20.4.2009; Steve J. Gregory leg.; garden around casa rurale Aztieta; ZMBN-ENT-PDESMID-185 • 1 ♂, ♀; Lekunberri, local exit N-130 direction to Betelu, 43.011N, 1.902W; ca. 580 m a.s.l.; 20.4.2009; Per Djursvoll leg.; industrial area, synantropic, meadow with stones and brick waste, under stones; ZMBN-ENT-PDESMID-145 • 4 ♂♂, 1 juvenile; Sierra de Aralar, south Baraibar, on road NA-7510 to Santuario de San Miguel; 42.9762N, 1.9318W; ca. 670 m a.s.l.; 22.4.2009; Per Djursvoll leg; under stones, in Corylus litter; ZMBN-ENT-PDESMID-142 • 1 ♀, Sierra de Urbasa, Alava, under northern border, on road A-2128 south of Opakua, 42.821N, 2.3549W; ca. 740 m a.s.l.; 23.4.2009; Steve J. Gregory leg.; woodland of Corylus, Quercus and Crategus; ZMBN-ENT-PDESMID-181 • 1 ♀; Sierra de Urbasa, on top at southern cliff border, east of road NA-7182, 42.7989N, 2.1417W; ca. 930 m a.s.l.; 23.4.2009; Steve J. Gregory leg.; pasture on stony ground, some thorny bushes, occasional trees or groups of *Fagus*; ZMBN-ENT-PDESMID-176. FRANCE – **Pyrénées-Atlantiques** • 2 ♂♂, 1 ♀, 2 juveniles; Tarnos; 43.5203N, 1.4639E, ca. 30 m a.s.l.; 26.4.2009; Desmond Kime & Per Djursvoll leg. mixed deciduous forest; ZMBN-ENT-PDESMID-143.

Notes. The species was described by Brolemann (1910) and is distributed in the French Pyrenees, and in northern Spain. Body length is 13–16 mm, the gonopods may resemble those of *Polydesmus inconstans* Latzel, 1884 (see Demange 1981: 125, figs 170– 171). It differs from *P. inconstans* in having a row of teeth dorsally on the endomere and if this character was not observed, probably misidentified as *P. inconstans* (Fig. 19) in the literature. It differs from species of the genus *Propolydesmus* with the presence of the well-developed exomere and endomere – with seminal groove and solenophore-pulvillus extended onto. These characters conform to those of the genus Polydesmus Latreille, 1802/03, and with its type species *Polydesmus complanatus* Linnaeus, 1761. *Propolydesmus racovitzai* is here transferred back to *Polydesmus*.

**Figures 17–20.**
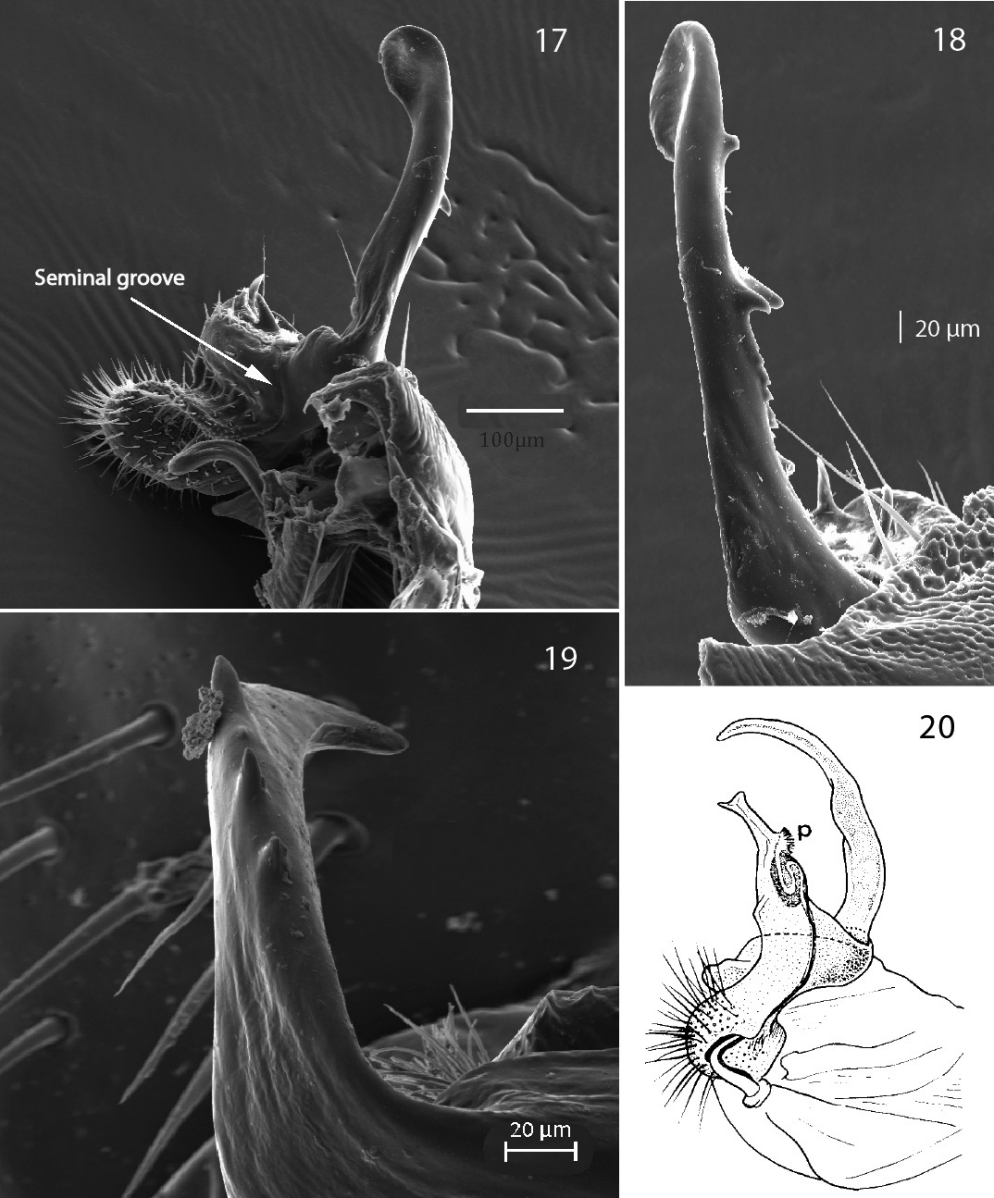
**17***Propolydesmus laevidentatus* (Loksa, 1967), from Madeira, right gonopod, medial view (ZMBN-ENT-PDESMID-342) **18***Propolydesmus laevidentatus* (Loksa, 1967), from Madeira, right gonopod, lateral view (ZMBN-ENT-PDESMID-342) **19***P. racovitzai* (Brolemann, 1910) right gonopod, lateral view (MNCN 20.07/1435) **20***P. haroi* (Mauriès & Vicente, 1977) gonopod, redrawn after Mauriès and Vicente (1977).

**Figures 21–24.**
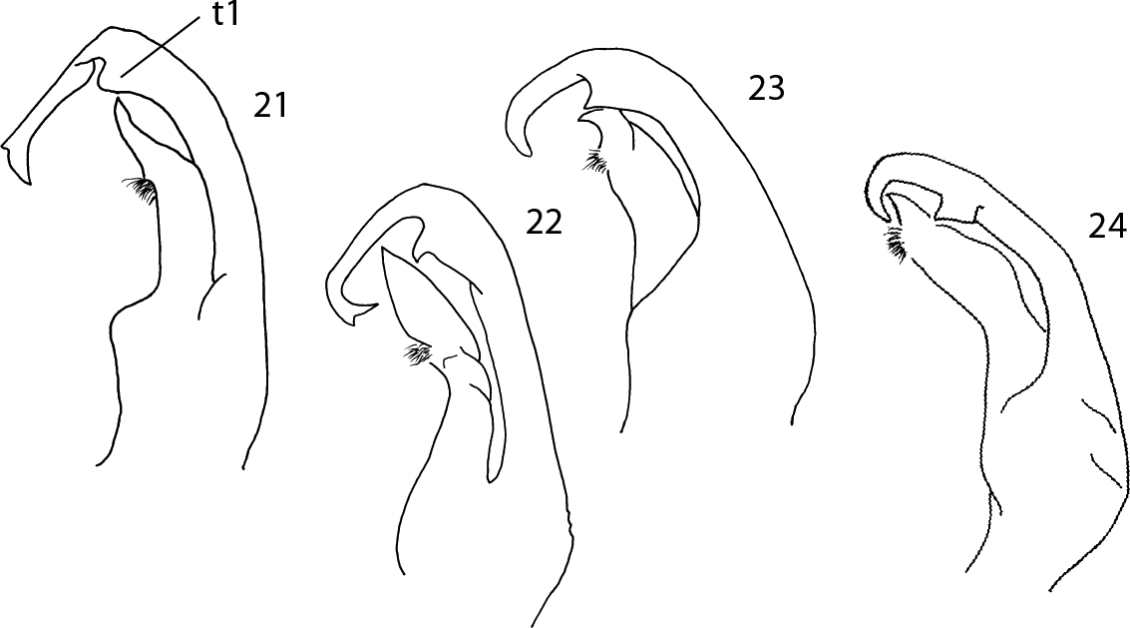
**21** Sketch of the gonopod of *P. angustus* Latzel, 1884 **22** Sketch of the gonopod of *P. incisus* Brolemann, 1921 **23** Sketch of the gonopod of *P. inconstans* Latzel, 1884 **24** Sketch of the gonopod of *P. coriaceus* Porat, 1871.

##### Key to the Iberian species of *Polydesmus*

**Table:** 

1	With 19 body rings in both sexes	***P. minutulus* Mauriès & Barraqueta, 1985**
–	With 20 body rings in both sexes	**2**
2	Gonopod unipartite, as in *Brachydesmus* Heller, 1858	***P. geochromus* Attems, 1952**
–	Gonopod bifurcate – exomere and endomere distinct	**3**
3	Exomere with a lateral tooth (t1) at main curvature point (Figs 21–24)	**4**
–	Exomere with a ventrolateral tooth (t1) at main curvature point (Figs 6, 11, 13–14)	**9**
4	T1-tooth at main curvature point on exomere quadrangular or blade-like (Fig. 24)	***P. coriaceus* Porat, 1871**
–	T1-tooth at main curvature point on exomere almost absent or triangular (Figs 21–23)	**5**
5	Endomere and exomere branches widely separated	***P. haroi* Mauriès & Vicente, 1977**
–	Endomere and exomere branches not widely separated	**6**
6	Endomere apically stout and blunt, somewhat hooked (Figs 23–24)	**7**
–	Endomere apically pointed – a1, not hooked (Figs 21–22)	**8**
7	With row of teeth dorsally on exomere (Fig. 19)	***P. racovitzai* Brolemann, 1910**
–	Without row of teeth dorsally on exomere	***P. inconstans* Latzel, 1884**
8	Acropodite narrow and acute (Fig. 21)	***P. angustus* Latzel, 1884**
–	Acropodite broad, leaf-shaped, with acute apex (Fig. 22)	***P. incisus* Brolemann, 1921**
9	Endomere with distinct cleavage, without acropodite a2 (Fig. 15)	***P. asturiensis* sp. nov.**
–	Endomere without distinct cleavage, with acropodites a1 and a2 (Fig. 4)	***P. biscayensi* s sp. nov.**

## Discussion

Enghoff and Golovatch (2003) redefined the small and solely southwestern European genus *Propolydesmus* Verhoeff, 1895, adding 12 species formerly placed in *Polydesmus* and adding one previously recognized *Propolydesmus* in synonymy, thus increasing the number of included species from four to 15. Consequently, the range of *Propolydesmus* has greatly expanded eastwards in Europe. Many of the species had been placed in subgenus Hormobrachium (Attems, 1940), that was synonymized with *Polydesmus* (s. str.) by Djursvoll et al. (2001). Later Djursvoll (2008) transferred *Propolydesmus mauriesi* (Vicente, 1979) to *Schizomeritius* Verhoeff, 1931. Without a species-level revision and a comprehensive analysis, the attributions of some of the species to *Propolydesmus* by Enghoff and Golovatch (2003) may be premature, as several of the species seem to have their affinities elsewhere.

Both Verhoeff (1895) and Djursvoll et al. (2001) in *Propolydesmus* diagnoses emphasized the character with particularly reduced endomere, while Enghoff and Golovatch (2003) stated “a relatively to very short/stout gonopod femorite”. This may have opened for the more extensive interpretation. However, Enghoff and Golovatch added for *Propolydesmus*, the important gonopodal character – presence of a seminal cavity, found in *Propolydesmus laevidentatus* (Loksa, 1967), in contrast to Verhoeff (1895) and Djursvoll et. al. (2001). In particular, *Propolydesmus laevidentatus*, as illustrated by Enghoff and Golovatch (2003: 83, figs 4–7) differs from *Polydesmus* by having a strongly reduced endomere, and a slightly curved exomere with numerous teeth (Figs 18, 19). In addition, a looped seminal groove that does not extend onto the endomere branch, also in accordance with the type species *Propolydesmus pectiniger* (Verhoeff, 1893).

## Supplementary Material

XML Treatment for Polydesmus biscayensis

XML Treatment for Polydesmus asturiensis

XML Treatment for Polydesmus haroi

XML Treatment for Polydesmus racovitzai
